# Relation of risk of contralateral breast cancer to the interval since the first primary tumour

**DOI:** 10.1038/sj.bjc.6605434

**Published:** 2009-11-17

**Authors:** C Rubino, R Arriagada, S Delaloge, M G Lê

**Affiliations:** 1 Institut National de la Santé et de la Recherche Médicale (INSERM), Unit 605, Villejuif, France; 2 Institut Gustave-Roussy (IGR), Villejuif, France; 3 Faculté de Médecine de Paris-Sud, Kremlin-Bicêtre, France; 4 Karolinska Institutet, Stockholm, Sweden

**Keywords:** breast cancer, natural history, contralateral breast

## Abstract

**Background:**

There is no consensus on how to separate contralateral breast cancer (CBC) occurring as distant spread of the primary breast cancer (BC) from an independent CBC.

**Methods:**

We used standardised incidence ratios (SIRs) to analyse the variations in the risk of CBC over time among 6629 women with BC diagnosed between 1954 and 1983. To explore the most appropriate cutoff to separate the two types of CBC, we analysed the deviance between models including different cutoff points as compared with the basal model with no cutoff date. We also performed a prognostic study through a Cox model.

**Results:**

The SIR was much higher during the first 2 years of follow-up than afterwards. The best cutoff appeared to be 2 years. The risk of early CBC was linked to tumour spread and the risk of late CBC was linked to age and to the size of the tumour. Radiotherapy was not selected by the model either for early or late CBC risk.

**Conclusion:**

A clearer pattern of CBC risk might appear if studies used a similar cutoff time after the initial BC.

The risk of a contralateral breast cancer (CBC) among breast cancer (BC) survivors has been estimated as two- to six-fold higher than that of the general population (Parker *et al*, 1989; Soerjomataram *et al*, 2005). However, variations in CBC risk according to the length of follow-up after the initial BC have not been clearly documented (Hankey *et al*, 1983; Brenner *et al*, 1993; Soerjomataram *et al*, 2005; Hooning *et al*, 2008). One study assessed variations in CBC risk by 5-year periods, but not by further details during the first 5 years (Hankey *et al*, 1983). Others exclusively analysed risk associated with asynchronous CBC, considered as a true independent CBC, whereas synchronous CBC were considered as tumour spread from the primary BC.

Currently, there is no consensus on how to distinguish independently from synchronous CBC. Indeed, the cutoff time separating them was arbitrarily chosen, varying widely between 0 and 12 months (Hislop *et al*, 1984; Basco *et al*, 1985; Bernstein *et al*, 1992; Broët *et al*, 1996; Chen *et al*, 2001; Gao *et al*, 2003; Hartman *et al*, 2005; Howe *et al*, 2005). The samples studied also varied because some studies selected only operable BC (Hankey *et al*, 1983; Broët *et al*, 1996), whereas others analysed populations selected using many criteria (Chen *et al*, 2001; Hartman *et al*, 2005; Hooning *et al*, 2008). It is probably for these reasons that the factors associated with the risk of asynchronous CBC differed between these studies.

To select the optimal cutoff time for separating independent from synchronous CBC, and also to identify the CBC risk factors associated with these categories, we analysed the variations in CBC risk according to the time elapsed since the first BC in a large cohort of patients treated between 1954 and 1983.

## Patients and methods

The initial cohort included 7711 invasive BC patients treated at the Institut Gustave-Roussy between 1954 and 1983. We excluded 882 women born abroad because of missing follow-up data, as most of them returned to their country of origin after treatment, and 200 who did not receive loco-regional treatment because they presented with distant metastases, or had other severe diseases. Thus, the study population comprised 6629 patients followed up until 31 December 2003, of whom only 4% were lost to follow-up; 78% had died by the end of the study. The mean follow-up was 24.7 years (s.e.=5.6) for the 1061 survivors. When deceased patients were included (as in Schemper and Smith, 1996), the median follow-up time was 10.6 years (95% confidence interval (CI): 0.7–21.3). We recorded CBC between the treatment of the first breast cancer and the first of the following dates: the last medical visit, death or 31 December 2003.

The analyses were based on the UICC (Union Internationale Contre le Cancer) TNM classification (UICC, 1974), the presence (yes/no) of inflammatory breast cancer (IBC), age (both continuous variables and three categories <40, 40–50 and 51 or more), calendar period (<1963, 1964–1973, 1974 or more), type of surgery (none, tumorectomy or mastectomy) and radiotherapy (yes/no). The radiation dose (mainly with Co60 units) most commonly used was 45 Gy in 18 fractions and 30 days, delivered to the chest wall (or the whole breast after breast-conserving surgery with a boost dose of 15 Gy in six fractions in 10 days, delivered to the tumour bed). All N+ patients also received lymph node radiotherapy to the axilla, supraclavicular area and internal mammary chain. Patients with N− axillary lymph nodes had not received lymph node irradiation. Less than 3% of the sample had received adjuvant chemo- or hormonotherapy, and therefore these parameters could not be studied. Our data included only four large tumour types: well-differentiated and undifferentiated adenocarcinomas, a combination of these two, and a pool of other histological types, including colloid, lobular and medullary carcinomas. This information was known for 96% of the patients who had undergone surgery, but it was not documented for other patients. All patients were regularly followed up at least every 6 months during the first 5 years and then yearly thereafter. A systematic annual mammography had been performed since the late 1950s.

### Statistical methods

The risk of CBC was analysed in two steps on the basis of external and internal comparisons. We compared the incidence of CBC in our cohort with that of BC in the general population using age-standardised incidence ratios (SIRs) estimated by Poisson regression. The reference rates were the national French BC incidence rates by 5-year age groups for 1975–1995 (Menegoz *et al*, 1997). For the periods before 1975 and after 1995, we took into account the general increase in breast cancer incidence in France, by age group, using linear regression and applying the regression coefficient observed in each 5-year period. Poisson regression was used to test the SIR for departure from unity. The 95% CI were calculated using maximum likelihood methods. To search for the most discriminating cutoff point between early and late CBC, we modelled CBC risk according to the different cutoff times since the first BC. Each model was nested in the baseline model with no cutoff, and the difference in deviance was estimated between these models and the baseline model, which follows a *χ*^2^ law with one degree of freedom; the greater the difference, the better the model explains the data regarding the change in the CBC risk along the time axis. We also analysed the relationships between the SIR and the covariates described above. We used a test of trend or heterogeneity for each variable and test of interaction between the SIR observed before and after the cutoff point; all tests were two-sided.

We performed a prognostic study, using CBC as the end point, through a Cox model (Cox, 1972), taking into account the cutoff time previously defined. We first performed a univariate analysis to select the covariates associated with CBC risk with a *P*-value of less than 0.25, which were analysed together in a multivariate model. We used AKAIKE information criterion to determine the best final model (Akaike, 1987). All analyses were stratified on the calendar period of first treatment.

## Results

The mean age was 56 years (range: 21–94 years), the largest part of the population showing a T2 (44%) and 18% a T3 or T4 tumour; 51% of the patients were classified N1, and 18% N2 or N3. Only 9% of the patients had initial metastases and 10% had IBC; 77% percent had undergone surgery, mostly a total mastectomy (64%), and 74% had received loco-regional radiotherapy. We recorded 673 CBCs that had occurred following a median time interval of 3 years with a wide range between 0 and 38 years. The 5-, 10-, 15- and 20-year rates of CBC were 7.1% (95% confidence interval (CI): 6.5–7.8), 10.5% (95% CI: 9.7–11.4), 12.8% (95% CI: 11.9–13.9) and 14.7% (95% CI: 13.6–15.9), respectively.

**Figure 1:**
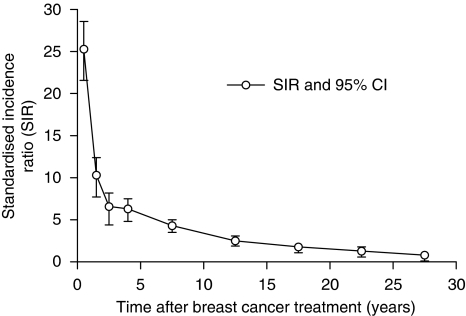
SIR of CBC between 0 and 30 years after the primary breast cancer.

Figure 1 shows the SIR of CBC according to the duration of follow-up after the diagnosis of the primary BC. Overall, the SIR was 4.7 (95% CI: 4.3–5.0). The SIR of CBC varied widely according to the length of the follow-up. Thus, a very high excess risk was observed during the first 2 years (25 in the first and 10 in the second year), compared with the general population. After 2 years, the risk decreased regularly until the 20th year, after which the CBC risk was not significantly different from that of the general population.

We found that the greatest difference in deviance between the basal model and models taking into account the different cutoff times corresponded to a cutoff time at 2 years of follow-up. This cutoff was therefore used for all further analyses, which included 275 early CBCs occurring within the first 2 years, and 398 late CBCs arising at 2 years or more.

The results shown in Table 1 include *P*-values, first for a test of trend or heterogeneity for each variable, and second for an interaction test between the SIR observed before and after 2 years. All the clinical characteristics of the primary breast cancer were linked to the risk of CBC for both early and late CBC. As the SIR was not significantly different between T0, T1 and T2 or between N0 and N1, these different tumour categories were pooled in the subsequent analyses. The excess risk for early CBC increased considerably with tumour extension. For instance, the SIR for patients with a T4 was 40.8 compared with 73.9 for patients with N3. For late CBC, similar increases in the SIR were observed, but to a lesser extent. Thus, the SIR for patients with a T4 was 7.5 compared with 8.6 for patients with N3. The differences between early and late CBC were clearly different for the N categories (test for interaction: *P*=0.02). An increased SIR was also evidenced for patients with distant metastases and IBC. No correlation was observed between any type of histology and the SIR of early and late CBC (data not shown).

There was clearly a higher risk of CBC for the younger (<40 years) patients for both early and late CBC. However, the risk of early CBC was 50-fold higher than that of the general population in the youngest patients, whereas the SIR was only 5-fold higher for late CBC. After the age of 40 years, the risk of early CBC was also higher than for late CBC. The interaction test (*P*=0.02) confirmed that the risk of CBC associated with age was different according to the time elapsed since the first BC (Table 2).

The CBC risks were higher for patients treated before 1973 than later. They were also much higher for early than for late CBC (interaction test *P*=0.002), because of a higher frequency of advanced primary tumours during the earliest periods (data not shown). For this reason, each model was stratified on the calendar period in the further internal comparison using a Cox model.

Patients who had not undergone surgery or who had received radiotherapy had a higher excess risk than the other patients. However, these parameters were strongly linked to tumour extension and their independent effect was studied in the multivariate analysis performed below.

All the variables described in Tables 1 and 2 were taken into account in the internal analysis, using a Cox model, stratified on the calendar period. The variables selected in the final models are shown in Table 3. The risk of early CBC was only associated with tumour extension and a more aggressive tumour (T, N and IBC). The risk of late CBC was essentially associated with age and UICC T. Neither radiotherapy nor surgery remained associated with the risk of early or late CBC, as the use of these treatments was strongly dependent on tumour extension. For both early and late CBC, no significant interaction was found between radiotherapy and age, even when the model was applied exclusively to the youngest women (<40 years old), or to 5-year survivors after the first BC treatment.

When the analyses were restricted to the 3752 breast cancer patients who had received surgery as the first treatment, similar results were observed (data not shown). Thus, the risk of early CBC was linked to a major prognostic factor in this category of patients, namely the number of involved lymph nodes (0, 1–3, 4–10, 11+). The risk of late CBC was only associated with age, and not with any other tumour extension parameter or type of treatment.

To see whether our results were altered when the cutoff time was changed, we performed additional analyses, using a cutoff time first at 6 months and second at 1 year. With these two cutoff times, we observed that all the factors found to be previously related to the risk of early CBC (T, N and IBC) were now associated with late CBC. Age and metastases were also linked to late CBC, whereas the risk of early CBC was only associated with UICC T.

Five-year survival rates were 38% (35–41%) after a CBC occurring during the first 2 years, and 56% (53–59%) after a later CBC. The corresponding 10-year survival rates were 26% (23–29%) and 42%, respectively (39–45%). Thus, the risk of death after CBC was 1.4-fold (1.2–1.7) higher for early CBC than for late CBC (*P*=10^−3^).

## Discussion

In this long-term cohort of 6629 breast cancer patients, we observed cumulative 10- and 20-year incidence rates of 10.5 and 14.7% for CBC similar to those of most previous reports (Hankey *et al*, 1983; Harvey and Brinton, 1985; Bernstein *et al*, 1992). We found that CBC risk was almost 5-fold higher than that of primary BC in the general population. This excess is probably overestimated because the systematic clinical and radiological follow-up of BC patients leads to earlier diagnosis, as in other studies (Hankey *et al*, 1983; Parker *et al*, 1989; Soerjomataram *et al*, 2005).

The CBC incidence ratios were not constant and varied along the time axis. They were much higher within the first 2 years after the first BC than later. After 20 years, the frequency of CBC was not significantly different from that of the general population. A similar decreased incidence ratio with the duration of follow-up has been reported (Obradovic *et al*, 1988; Schwartz *et al*, 1989; Brenner *et al*, 1993). In other studies, this was only observed in certain subgroups, such as those with lymph node involvement (Hankey *et al*, 1983), or among the youngest women (Hartman *et al*, 2005). These variations are probably partly explained by the selection of the populations studied, which mostly excluded patients with a high risk of early CBC.

In our study, conducted on an unselected population, we showed that 2 years was the most appropriate cutoff to separate CBC occurring as potential tumour spread from the primary BC from independent CBC. A single cutoff time is probably not the best model for the variation in overall incidence of CBC over time. Other more complex models might be considered. However, for a clinical approach, a single cutoff appears to be the most adequate way to discriminate the spread dependent on a primary BC from that on an independent CBC. One of the major limits of this method is that there is a mixed population of the two types of CBC around the cutoff point. However, the fact that the risk of death was very different between early CBC and late CBC indicates that this cutoff point accurately separates these two distinct entities.

Regarding the initial tumour characteristics (Table 1), both early and late CBC were associated with more advanced disease, including IBC, even if a significant interaction was only found for the clinical N. However, in the multivariate analysis (Table 3), early CBC risk was strongly dependent on tumour extension, whereas late CBC risk was, apart from age, only associated with clinical tumour size (T). This last result suggests that at least some of the early CBC may be part of a generalised metastatic process and that late CBC comprises two types – independent CBC associated with age and CBC reflecting later metastatic dissemination. Similar results were observed in part of our sample, which included patients treated at the Institut Gustave-Roussy in 1967–1972 (Fontaine *et al*, 1986). In an unselected population, patients with early CBC (occurring 3 months after first BC) had a locally advanced or metastatic first BC more often than patients with CBC occurring between 3 and 60 months, or in those with no CBC (Howe *et al*, 2005).

Two reports failed to show a relationship between CBC risk and tumour extension (Bernstein *et al*, 1992; Broët *et al*, 1996), but chemotherapy was administered to a large proportion of subjects, which exerted a protective effect on CBC risk. Hence, this might have cancelled the effect of tumour extension. Our patients received no chemotherapy, and this may have allowed the natural history of CBC to be more apparent.

The cutoff point at 2 years contrasts with the commonly assumed cutoffs of 6 months or less (Bernstein *et al*, 1992; Broët *et al*, 1996; Chen *et al*, 2001; Gao *et al*, 2003; Hartman *et al*, 2005; Howe *et al*, 2005). We therefore repeated our analysis with a cutoff at 6 months to see whether our results changed. The results showed that a considerable portion of the previous early CBC became ‘new’ late CBC. These latter CBC were therefore linked to the extension of the first BC (TNM, IBC). The ‘new’ early CBC were only associated with UICC T, suggesting that CBC occurring before 6 months might correspond to the local extension of initially large tumours rather than a CBC reflecting metastatic dissemination.

In the external analysis, we observed a strong relationship between an early age and increased risk of CBC, for both early and late CBC (Table 2). However, when a multivariate analysis was performed (Table 3), age remained associated with the risk of CBC only for late CBC, the relationship with risk of early CBC being entirely explained by more aggressive lesions and tumour spread occurring in younger patients. Our findings on late CBC risk have often been reported (Hankey *et al*, 1983; Kurtz *et al*, 1988; Obradovic *et al*, 1988; Brenner *et al*, 1993; Broët *et al*, 1996; Mariani *et al*, 1997; Chen *et al*, 2001; Hartman *et al*, 2005; Mellemkjaer *et al*, 2006). In a study that did not analyse women above the age of 54 years such a relationship was not found (Bernstein *et al*, 1992). In another study, women aged 56 or more had a higher risk of CBC than women aged 45–55 years, but a similar risk to that of the youngest women (<45) (Gao *et al*, 2003). Here, however, a cutoff time of 3 months was used, and probably included many patients with synchronous CBC, which was not associated with age in this study.

In the external analysis, for both early and late CBC, the risk was higher during the early years of treatment than during more recent years, although less so for late CBC (Table 2). As the calendar periods are strongly associated with tumour extension, the Cox models were stratified on this parameter. Our study included BC patients diagnosed during 1954–1983, when the frequency of locally advanced BC was much higher than it is today. We therefore verified our results among the operable BC that included more than 3000 patients. We found that a cutoff time of 2 years was still valid to discriminate synchronous from independent CBC. Indeed, we found that the factor most strongly associated with the risk of early CBC was the number of involved lymph nodes, which is a major prognostic factor (Chin and Guerra, 1980; Lee and Chan, 1984). The only factor associated with the risk of late CBC was age. These findings suggest that the cutoff time of 2 years is highly relevant for separating synchronous CBC, occurring as potential spread of the primary tumour, from independent CBC, even among patients with early BC.

Treatment indications are strongly related to initial tumour extension. For instance, patients with inoperable BC or those treated with radiotherapy, who had greater tumour extension, incurred a higher risk of CBC than other patients in the external analysis (Table 2). However, in the multivariate analysis, neither the type of surgery nor radiotherapy remained associated with the risk of CBC. However, our study was not powerful enough to show a possibly small radiotherapy effect. Only randomised trials on adjuvant radiotherapy are theoretically able to measure, devoid of bias, the carcinogenic effect of radiation therapy among women treated for BC. A recent review of these trials (EBCTCG, 2005a) showed that the risk of CBC among women with early BC was slightly higher among the irradiated patients than among the non-irradiated patients. However, these results merit discussion for two reasons: first, this excess risk exists only between 5 and 14 years of follow-up and not later, and second, the risk is higher for older (after 50 years) than for younger women. After the first few years of follow-up, a differential quality of follow-up might occur between the irradiated and non-irradiated women, resulting from other possible adverse effects of radiotherapy. For instance, patients with any radiation complication, such as arm lymphoedema, are more likely to have complete screening, including mammographies of the contralateral breast, than women without complications, thus introducing a potential follow-up bias.

Most reports have not shown a relationship between the risk of CBC and radiotherapy (Basco *et al*, 1985; Parker *et al*, 1989; Bernstein *et al*, 1992; Broët *et al*, 1996; Gao *et al*, 2003; Soerjomataram *et al*, 2005; Hooning *et al*, 2008), although some found that the potential risk was higher for the youngest women, after a long follow-up, and/or for those who had received a higher radiation dose to the contralateral breast (Boice *et al*, 1992; Hooning *et al*, 2008; Stovall *et al*, 2008). These results are in conformity with previous reports on BC risks among women who received radiotherapy during 1930–1970 for various benign disorders, in which this decreased markedly with age at radiation exposure (Shore *et al*, 1986; Tokunaga *et al*, 1987; Preston *et al*, 2002; UNSCEAR, 2008).

A decreased risk of CBC has been reported as being associated with adjuvant treatments (Bernstein *et al*, 1992; Broët *et al*, 1996; EBCTCG, 2005b; Bertelsen *et al*, 2008; Yadav *et al*, 2008); most patients in our study did not receive chemotherapy or hormone therapy.

Our study failed to show any relationship between the tumour histological type and CBC risk, although our registered data set was not sufficiently detailed to clarify this further. Numerous authors found an association between the risk of CBC and lobular carcinoma (Horn *et al*, 1987; Horn and Thompson, 1988; Bernstein *et al*, 1992; Broët *et al*, 1996; Cook *et al*, 1996), but Hislop *et al* (1984), found such a relationship exclusively for early CBC (occurring within the first year after the first BC), and not for later CBC. Gao *et al* (2003), showed a higher risk of CBC in association with medullary carcinoma. These disparities may reflect the choice of cutoff time for identifying synchronous and asynchronous CBC.

In conclusion, CBC is a common disease after a first BC, and involves a regular clinical and mammographic follow-up.

Early onset of CBC was associated with a worse prognosis, but clearer patterns of CBC risk might appear if studies used a similar cutoff time after the first diagnosis. Our study suggests that this should be at 2 years after first BC diagnosis.

## Figures and Tables

**Table 1:** Standardised incidence ratios of contralateral breast cancer relative to the French population according to the TNM UICC characteristics of the primary BC and time elapsed since the first BC

		**Cases**					
**Characteristics and time since the first BC**	**PYR**	**Observed**	**Expected**	**SIR**	**95% CI**	*P*-value*	*P*-value**
*T UICC*							
<2 years							
0	166	3	0.2	12.5	4.0–38.7	<10^−3^	
1	2108	31	3.0	10.4	7.3–14.7		
2	5494	79	7.3	10.8	8.7–13.5		
3	2241	61	2.4	25.9	20.1–33.2		
4	1577	91	2.3	40.8	33.2–50.1		
⩾2 years							0.18
0	1150	8	2.6	3.1	1.6–6.2	<10^−3^	
1	15443	88	34.2	2.6	2.1–3.2		
2	32825	170	66.8	2.5	2.2–3.0		
3	9205	71	16.3	4.4	3.5–5.5		
4	2922	45	6.0	7.5	5.6–10.1		
							
*N UICC*							
<2 years							
0	4405	67	6.5	10.4	8.2–13.2	<10^−3^	
1	6173	126	7.5	16.8	14.1–20.0		
2	858	48	0.9	54.2	40.8–71.9		
3	403	34	0.5	73.9	52.8–103.5		
⩾2 years							0.02
0	27178	156	59.3	2.6	2.2–3.1	<0.01	
1	33578	214	65.5	3.3	2.9–3.7		
2	2049	20	3.3	6.1	3.9–9.5		
3	545	8	0.9	8.6	4.3–17.2		
							
*M UICC*							
<2 years							
0	11280	241	14.7	16.4	14.4–18.6	<10^−3^	
1	560	34	0.7	46.9	33.5–65.6		
⩾2 years							0.6
0	62844	389	128.3	3.0	2.7–3.3	0.02	
1	506	9	0.9	10.5	5.4–20.1		
							
*Inflammatory BC*							
<2 years							
No	10940	212	14.2	14.9	13.0–17.1	<10^−3^	
Yes	900	63	1.2	51.5	40.2–65.9		
⩾2 years							0.12
No	61736	375	125.8	3.0	2.7–3.3	<0.01	
Yes	1614	23	3.3	6.9	4.6–10.5		

Abbreviations: CI=Confidence interval; PYR=Number of person-years at risk; SIR=Standardised Incidence Ratio; UICC=Union internationale contre le cancer. The SIR is equal to the ratio between observed and expected cases during the follow-up. ^*^Test of trend for quantitative variables, test of heterogeneity for qualitative variables; ^**^Interaction test between BC characteristics and the duration of follow-up.

**Table 2:** Standardised incidence ratios of contralateral breast cancer according to age at treatment, calendar period of treatment, types of treatment and time elapsed since the first BC

		**Cases**					
**Characteristics and time since the first BC**	**PYR**	**Observed**	**Expected**	**SIR**	**95% CI**	***P*-value**	***P*-value**
*Age (years)*							
<2 years							
<40	1138	26	0.5	50.8	34.6–74.6	<10^−3^	
40–50	3008	74	3.6	20.6	16.4–25.9		
>50	7694	175	11.2	15.6	13.5–18.1		
⩾2 years							0.02
<40	7076	62	12.0	5.2	4.0–6.6	<10^−3^	
40–50	21 071	119	43.4	2.7	2.3–3.3		
>50	35 203	217	73.7	2.9	2.6–3.4		
							
*Calendar period*							
<2 years							
<1963	2289	61	1.6	38.2	29.8–49.3	<10^−3^	
1963–1973	3377	85	3.9	21.9	17.7–27.1		
>1973	6174	129	9.8	13.1	11.0–15.6		
⩾2 years							0.002
<1963	12 006	69	17.7	3.9	3.1–4.9	0.02	
1963–1973	18 169	120	35.2	3.4	2.8–4.1		
>1973	33 175	209	76.1	2.7	2.4–3.1		
							
*Surgery*							
<2 years							
No surgery	2135	106	2.9	36.0	29.7–43.5	<10^−3^	
Tumorectomy	1681	22	2.6	8.5	5.6–12.9		
Mastectomy	8024	147	9.8	15.0	12.8–17.7		
⩾2 years							0.08
No surgery	4066	49	7.7	6.3	4.8–8.4	<10^−3^	
Tumorectomy	11 393	73	26.3	2.8	2.2–3.5		
Mastectomy	47 891	276	94.9	2.9	2.6–3.3		
							
*Radiotherapy*							
<2 years							
No	3266	47	4.3	11.0	8.3–14.6	<10^−3^	
Yes	8574	228	11.0	20.7	18.1–23.5		
⩾2 years							0.15
No	23254	116	47.5	2.4	2.0–2.9	0.01	
Yes	40096	282	81.5	3.5	3.1–3.9		

Abbreviations: CI=Confidence interval; PYR=Number of person-years at risk; SIR=Standardised Incidence Ratio. The SIR is equal to the ratio between observed and expected cases during the follow-up. ^*^Test of trend for quantitative variables, test of heterogeneity for qualitative variables. ^**^Interaction test between each variable and the duration of follow-up.

**Table 3:** Prognostic factors for CBC in multivariate analyses using a Cox model

**Time elapsed since the first BC**	**Risk factors**	**Number of events/total**	**RR (95% CI)**	*P*-value*
<2 years				
	*T UICC*			
	0–2	113/4098	1^a^	<10^−3^
	3	61/1312	1.4 (1.0–2.0)	
	4	101/1219	2.1 (1.5–3.0)	
				
	*N UICC*			
	0–1	193/5727	1^a^	<10^−3^
	2	48/585	1.8 (1.3–2.5)	
	3	34/317	2.3 (1.5–3.4)	
				
	*Inflammatory BC*			
	No	212/5995	1^a^	
	Yes	63/634	1.6 (1.2–2.3)	<0.01
				
⩾2 years				
	*Age in years*			
	<40	68/596	1.4 (1.1–1.8)	<0.01
	40–50	121/1452	1.0 (0.8–1.2)	
	>50	209/3197	1^a^	
				
	*T UICC*			
	0–2	266/3645	1^a^	<10^−3^
	3	71/930	1.4 (1.1–1.8)	
	4	61/670	2.2 (1.6–2.9)	

Abbreviations: CI=Confidence interval; UICC=Union internationale contre le cancer;

* Test of trend for quantitative variables, test of heterogeneity for qualitative variables.

a Reference category.
